# Involvement of gut microbial fermentation in the metabolic alterations occurring in n-3 polyunsaturated fatty acids-depleted mice

**DOI:** 10.1186/1743-7075-8-44

**Published:** 2011-06-27

**Authors:** Barbara D Pachikian, Audrey M Neyrinck, Laurence Portois, Fabienne C De Backer, Florence M Sohet, Myrjam Hacquebard, Yvon A Carpentier, Patrice D Cani, Nathalie M Delzenne

**Affiliations:** 1 Metabolism and Nutrition Research Group, Louvain Drug Research Institute, Université catholique de Louvain, Brussels, Belgium; 2 Laboratory of Experimental Surgery, Université Libre de Bruxelles, Brussels, Belgium

**Keywords:** n-3 polyunsaturated fatty acid, gut microbiota, prebiotic, energy efficiency

## Abstract

**Backround:**

Western diet is characterized by an insufficient n-3 polyunsaturated fatty acid (PUFA) consumption which is known to promote the pathogenesis of several diseases. We have previously observed that mice fed with a diet poor in n-3 PUFA for two generations exhibit hepatic steatosis together with a decrease in body weight. The gut microbiota contributes to the regulation of host energy metabolism, due to symbiotic relationship with fermentable nutrients provided in the diet. In this study, we have tested the hypothesis that perturbations of the gut microbiota contribute to the metabolic alterations occurring in mice fed a diet poor in n-3 PUFA for two generations (n-3/- mice).

**Methods:**

C57Bl/6J mice fed with a control or an n-3 PUFA depleted diet for two generations were supplemented with prebiotic (inulin-type Fructooligosaccharides, FOS, 0.20 g/day/mice) during 24 days.

**Results:**

n-3/-mice exhibited a marked drop in caecum weight, a decrease in lactobacilli and an increase in bifidobacteria in the caecal content as compared to control mice (n-3/+ mice). Dietary supplementation with FOS for 24 days was sufficient to increase caecal weight and bifidobacteria count in both n-3/+ and n-3/-mice. Moreover, FOS increased lactobacilli content in n-3/-mice, whereas it decreased their level in n-3/+ mice. Interestingly, FOS treatment promoted body weight gain in n-3/-mice by increasing energy efficiency. In addition, FOS treatment decreased fasting glycemia and lowered the higher expression of key factors involved in the fatty acid catabolism observed in the liver of n-3/-mice, without lessening steatosis.

**Conclusions:**

the changes in the gut microbiota composition induced by FOS are different depending on the type of diet. We show that FOS may promote lactobacilli and counteract the catabolic status induced by n-3 PUFA depletion in mice, thereby contributing to restore efficient fat storage.

## Background

Dietary n-3 polyunsaturated fatty acids (PUFA) have received considerable attention during the last decades. Many epidemiological and clinical studies have described the beneficial effects of these fatty acids on cardiovascular disease [1], inflammation [2,3], insulin sensitivity [4,5] and hypertriglyceridemia [6]. In addition, by their ability to modulate the expression of regulatory genes, n-3 PUFA can coordinate an upregulation of lipid oxidation and a downregulation of lipid synthesis [6-8].

However, a decrease in n-3/n-6 PUFA ratio is observed in western diets [9,10] and is well known to be associated with an increased risk of cardiovascular disease and inflammation [9,11]. Moreover, studies have described a lower level of n-3 PUFA in plasma [12,13], in liver and in erythrocyte phospholipids [14] of obese people and an lower n-3/n-6 PUFA ratio in the hepatic phospholipids of patients with non alcoholic fatty liver disease [15]. Several data have demonstrated that n-3 PUFA depleted rats exhibit some features of the metabolic syndrome including visceral obesity [16], hepatic steatosis [17], insulin resistance [18], cardiac hypertrophy [19] and perturbation of metabolic, ionic and functional events in pancreatic islets [18,20].

In a previous study, we have shown that mice fed with a diet poor in n-3 PUFA during two generations (n-3/- mice) exhibit a depletion in n-3 PUFA in both hepatic phospholipids and triglycerides (TG) fraction [21]. Moreover, n-3 PUFA depleted mice show a decreased body weight despite similar caloric intake, a higher fasting glycemia and triglyceride accumulation (steatosis) in the liver [21]. The hepatic mRNA content of fatty acid synthase (FAS)-the rate-limiting enzyme for lipid synthesis-was decreased, whereas carnitine palmitoyl transferase 1 (CPT1) and peroxisome proliferator-activated receptor gamma coactivator α (PGC1α)-two key factors involved in β-oxidation-were increased, suggesting catabolic alterations of the energy homeostasis in the liver tissue of n-3 PUFA depleted mice [21].

The microbial fermentation of nutrients is an important parameter to take into account when considering energy balance [22,23]. Both dietary fat and fermentable carbohydrates modulate the gut microbiota composition and the extent of fermentation [24]. Carbohydrates that partially or totally resist the digestion in the upper part of the gut may be fermented by the saccharolytic colonic bacteria, with consequences on host energy harvest and storage [22,25]. Moreover, some carbohydrates exhibit interesting nutritional properties, linked to their preferential use by specific types of bacteria. This is the case of inulin-type fructans with prebiotic properties that allow specific changes both in the composition and or the activity of the gastrointestinal microbiota in favour of bifidobacteria [26-28], thereby decreasing inflammation and metabolic disorders -including steatosis- in obese mice [29]. An increase in total fat intake promotes fat mass development at a higher extent in mice with a complex gut microbiota than in germ-free mice, suggesting a key role played by bacteria on host energy storage [30]. Among saccharolytic bacteria, some of them have been proposed to be involved in fat storage in certain conditions and models. The increase in Firmicutes has been associated with an increase in fat storage upon obesity [31,32]. Some strains of lactobacilli have been shown to increase body weight in chicken [33], whereas other strains rather decrease fat mass in mice models of obesity or in humans [34]. On the other hand, the consumption of a high fat diet profoundly modifies mouse gut microbiota composition, leading namely to a decrease in bifidobacteria and bacteroides [24,35]. The effects of the qualitative modulation of dietary fat content on gut microbiota and their consequences on metabolic disorders remain largely unexplored.

We have previously observed that mice born from a mother fed a diet depleted in n-3 PUFA, and maintained on this type of diet for several months, exhibit a decrease in caecal tissue and content weights, suggesting a lower caecal fermentation, as compared to mice fed a standard diet [21]. Therefore, this study aimed at analyzing in the same model of nutritional n-3 PUFA depletion, the relevance of gut microbiota in the onset of metabolic disorders in n-3/- mice. For this purpose, n-3/- or n-3/+ mice were treated during 24 days with prebiotics (FOS). Some results were partly described in our previous article [21], only the two groups of FOS treated mice were added in this current study.

## Materials and methods

### Animals and diets

Old (aged from 33 to 35 weeks) female C57Bl/6J mice fed a control diet (n-3/+, n = 9) or treated for two generations with a diet containing sub-minimal amount of n-3 PUFA (n-3/-, n = 12) were housed at 22°C in an 12 h light/dark cycle and given free access to diet and water. The control diet (n-3/+ diet: AO3, SAFE, Villemoison-sur-orge, France) contained the following (percent w/w): protein 21, digestible carbohydrates 52 (including starch 34 and saccharose 3.8), soya oil 5, cellulose 4, vitamin and mineral mixture 5 and water 12. The n-3/- diet was obtained by the use of sunflower oil which present a lower n-3/n-6 PUFA ratio compared with the soya oil used for the n-3/+ diet. The n-3/- diet contained the following (percent; w/w): protein 23, digestible carbohydrates 62 (corn starch 36 and saccharose 26), sunflower oil 5, agar-agar 2, cellulose 2, vitamin mixture 5 and mineral mixture 1. The n-3/n-6 ratio was 0.164 and 0.08 for the control diet and the n-3 PUFA-depleted diet, respectively. The detailed fatty acid pattern of these diets was fully described previously [18]. A group of n-3/+ (n-3/+ FOS; n = 10) and n-3/- mice (n-3/- FOS; n = 12) were supplemented for 24 days with FOS (gift from Orafti; Tienen, Belgium). FOS was added to tap water in a concentration adequate to reach an intake of 0.2 g of FOS per day. The energy content of the four diets is 331 kcal/100 g for n-3/+, 346 kcal/100 g for n-3/+ FOS, 385 kcal/100 g for n-3/- and 400 kcal/100 g for n-3/- FOS.

All mouse experiments were approved by the local animal ethics committee and the housing conditions were as specified by the Belgian Law of November 14, 1993 on the protection of laboratory animals (agreement n LA 1230314).

### Food intake assessment

Food intake, taking into account spillage, was recorded twice weekly during the last three weeks.

### Oral glucose tolerance test

An oral glucose tolerance test (gavage with 3 mg glucose/g body weight; 66% glucose solution) was performed on 6 h-fasted mice one week before the end of the treatment. Blood glucose was determined with a glucose meter (Roche diagnostic) on 3.5 μl of blood collected from the tip of the tail vein, 30 min before and 0, 15, 30, 60, 90 and 120 min following glucose injection. Insulin was measured in 5 μl of plasma samples obtained from tail blood at -30 and 15 min using an ELISA kit (Mercodia, Upssala, Sweden). The insulinogenic index was defined as the ratio of the difference between plasma insulin concentrations (pM) at 0 and 15 min to the difference between blood glucose concentrations (mM) at 0 and 15 min. The homeostasis model assessment of insulin resistance was calculated as [fasted glycemia (mM)*fasted insulinemia (μU/ml)]/22.5.

### Tissue samples

At the age of 34 ± 1 weeks, mice were anaesthetized by intra-peritoneal injection of sodium pentobarbital solution (Nembutal_®_, 60 mg/kg of body weight, Sanofi Santé Animale, Benelux, Brussels). The liver tissue was immediately clamped in liquid N_2 _and kept at -80°C. Caecal content and proximal colon were collected and stored at -80°C. Liver, fat tissues (ovarian, subcutaneous, and visceral), caecal content and caecal tissue were weighed.

### Blood biochemical analysis

Vena cava blood samples were collected in EDTA tubes. After centrifugation (10 min at 1500 g), plasma was stored at -80°C. Plasma TG and cholesterol (Elitech diagnostics, Sees, France) concentrations were measured using kits coupling enzymatic reaction and spectrophotometric detection of reaction end-products.

### Tissue biochemical analysis

For hepatic lipid content measurement, 100 mg of liver tissue was homogenized in 0.9 ml of phosphate buffer (pH 7.4). Lipids were extracted by mixing 125 μl of homogenate with 1 ml of 2:1 chloroform: methanol [36]. The chloroform phase was evaporated under nitrogen flux, and the dried residue was solubilized in isopropanol. TG or cholesterol were measured as previously described for plasma samples.

### Real-time quantitative PCR

Total RNA was isolated from liver tissue and proximal colon (Roche Diagnostics Belgium, Vilvoorde). cDNA was prepared by reverse transcription of 1 μg total RNA using the Kit Reverse transcription System (Promega, Leiden, The Netherlands). Real-time PCRs were performed with StepOnePlus™ Real-time PCR system (Applied Biosystems, Foster City, CA, USA) using SYBER-Green for detection. Ribosomal protein L19 (RPL19) RNA was chosen as an invariant standard. The primers used were as follows: for RPL19, sense, 5'-GAAGGTCAAAGGGAATGTGTTCA-3'; antisense, 5'-CCTTGTCTGCCTTCAGCTTGT-3'; for FAS, sense, 5'-TTCCAAGACGAAAATGATGC-3'; antisense, 5'-AATTGTGGGATCAGGAGAGC-3'; for CPT1, sense, 5'-AGACCGTGAGGAACTCAAACCTAT-3'; antisense, 5'-TGAAGAGTCGCTCCCACT-3'; for peroxisome proliferator-activated receptor α (PPARα), sense, 5'-CAACGGCGTCGAAGACAAA-3'; antisense, 5'-TGACGGTCTCCACGGACAT-3'; for PGC1α, sense, 5'-AGCCGTGACCACTGACAACGAG-3'; antisense, 5'-GCTGCATGGTTCTGAGTGCTAAG-3'; for proglucagon, sense, 5'-ATGAAGACCATTTACTTTG-3'; antisense, 5'-CGGTTCCTCTTGGTGTTCATCAAC-3'. All tissues were run in duplicate in a single 96-well reaction plate (MicroAmp Optical, Applied Biosystems) and data were analysed according to the 2-ΔCT method. The identity and purity of the amplified product were checked through analysis of the melting curve carried out at the end of amplification.

### Real-time quantitative PCR for microbial caecal content

The QIAamp DNA Stool Minikit (Qiagen) was used to extract DNA from caecal content according to the manufacturer's instructions. The primers and probes used to detect *Bifidobacterium *spp., *Lactobacillus *spp., and *Bacteroides-Prevotella *were based on 16S rRNA gene sequences as described previously [37]. The PCR amplification reactions were carried out as follows; 2 min at 50°C, 10 min at 95°C, followed by 45 cycles of 15 s at 95°C and 1 min at 60°C, and detection was carried out on a StepOnePlus™ Real-time PCR system (Applied Biosystems, Foster City, CA, USA). Each assay was performed in duplicate in the same run. The cycle threshold of each sample was then compared to a standard curve made by diluting genomic DNA (5-fold serial dilution) from cultures of *Bifidobacterium Longum, Lactobacillus acidophilus *and *Bacteroides fragilis*. Cell counts before DNA extraction were determined with the Neubauer hemocytometer. To determine the sensitivity and specificity of the assays, the PCR assays were confirmed by using a set of intestinal bacterial species as controls. Primers used were as follows: *Bifidobacterium*, sense, CGCGTCYGGTGTGAAAG; antisense, CCCCACATCCAGCATCCA; *Lactobacillus*, sense, GAGGCAGCAGTAGGGAATCTTC; antisense, GGCCAGTTACTACCTCTATCCTTCTTC; *Bacteroides-Prevotella*, sense, GAGAGGAAGGTCCCCCAC; antisense, CGCTACTTGGCTGGTTCAG. Taqman probes were as follows: BHQ-1-bifido, AACAGGATTAGATACCC; BHQ-1-lacto, ATGGAGCAACGCCGC; *Bacteroides-Prevotella*, VIC-CCATTGACCAATATTCCTCACTGCTGCCT-TAMRA [38].

### Statistical analysis

Results are presented as mean ± SEM. Statistical significance between groups was assessed by Student *t*-test using GraphPad Prism version 4.00 for Windows. *P <*0.05 was considered as statistically significant.

## Results

### Prebiotic intervention increases the caecal fermentation and intestinal proglucagon expression in both groups of mice but differentially modulates the gut microbiota in n-3/- and n-3/+ mice

**Figure 1:**
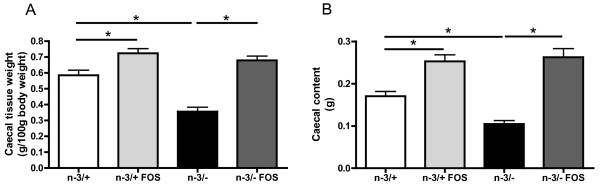
**Caecal content and tissue weight**. (A) caecal tissue weight (g/100 g body weight), (B) caecal content weight (g). Data are the mean ± SEM. *: mean values significantly different (P < 0.05, Student's *t*-test). n-3/+: mice fed with a control diet (n = 9), n-3/+ FOS: mice fed a control diet and supplemented with a prebiotic for 24 days (n = 10), n-3/-: mice fed an n-3 PUFA-depleted diet (n = 12), n-3/- FOS: mice fed an n-3 PUFA-depleted diet and supplemented with a prebiotic for 24 days (n = 12). The results have been partly reported in reference 20.

n-3/- mice have a low caecal tissue and content weights compared with n-3/+ mice. FOS supplementation increases caecal tissue and content weights in both groups of mice (Figure 1A and 1B).

**Figure 2:**
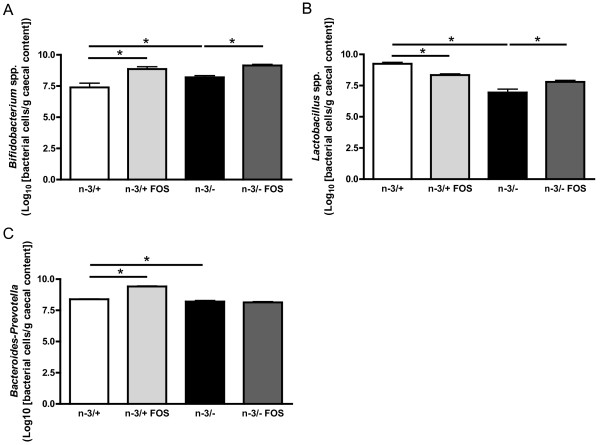
**Microbial caecal content**. (A) *bifidobacterium *spp., (B) *lactobacillus *spp., (C) *bacteroides-prevotella*. Bacterial quantities are expressed as Log_10 _(bacterial cells/g caecal content). Data are the mean ± SEM. *: mean values significantly different (P < 0.05, Student's *t*-test). -3/+: mice fed with a control diet (n = 9), n-3/+ FOS: mice fed a control diet and supplemented with a prebiotic for 24 days (n = 10), n-3/-: mice fed an n-3 PUFA-depleted diet (n = 12), n-3/- FOS: mice fed an n-3 PUFA-depleted diet and supplemented with a prebiotic for 24 days (n = 12).

Lactic acid producing bacteria, namely bifidobacteria and lactobacilli have been quantified in the caecal content of mice. Surprisingly, as shown in Figure 2, the bifidobacteria level was higher in n-3/- mice than in n-3/+ mice. FOS supplementation in n-3/+ and n-3/- mice lead to 9 to 30 fold higher caecal content in bifidobacteria, confirming the bifidogenic effect already observed in several mice models. Lactobacilli basal level was lower in n-3/- mice than in n-3/+ mice. FOS treatment decreased the content of lactobacilli in n-3/+ mice whereas it increased the lactobacilli in n-3/- mice (Figure 2B). The level of *Bacteroides-Prevotella*, an important family of gram negative bacteria, was slightly (1.5 fold) lower in n-3/-mice than in n-3/+ mice. FOS treatment promoted bacteroides in n-3/+ mice but has no effect in n-3/- mice (Figure 2C).

The prebiotic effect of FOS is reflected through its capacity to increase the expression of proglucagon gene in the colon [39].

**Figure 3:**
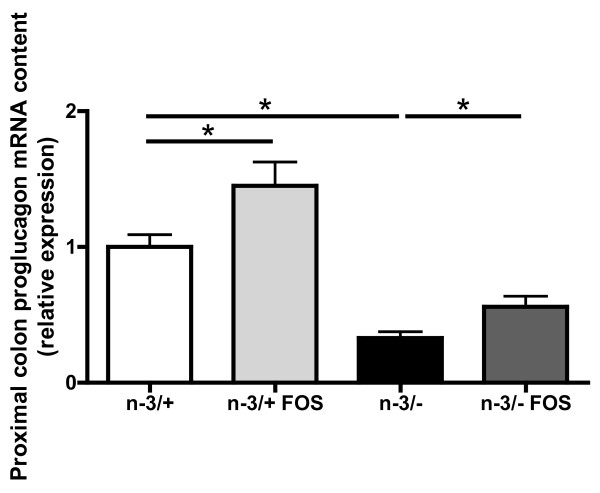
**Proximal colon proglucagon mRNA content**. Data are the mean ± SEM. *: mean values significantly different (P < 0.05, Student's *t*-test). -3/+: mice fed with a control diet (n = 9), n-3/+ FOS: mice fed a control diet and supplemented with a prebiotic for 24 days (n = 10), n-3/-: mice fed an n-3 PUFA-depleted diet (n = 12), n-3/- FOS: mice fed an n-3 PUFA-depleted diet and supplemented with a prebiotic for 24 days (n = 12).

The beneficial effects of FOS supplementation on the improvement of glucose metabolism are partly linked to an increased production of glucagon-like peptide-1 [40]. This peptide is produced through post-translational modification of the proglucagon mRNA. Proglucagon mRNA content in the proximal colon was lower in n-3/- than in n-3/+ mice. As expected, FOS supplementation increased proglucagon mRNA content in both groups of mice, leading to a nearly two-fold increase in proglucagon mRNA expression in n-3/- mice (Figure 3).

#### Prebiotic intervention increases body weight gain and energy efficiency in n-3/- and n-3/+ mice

Body weight evolution during the follow-up was different in n-3/- mice and in n-3/+ mice (data not shown). FOS supplementation increased body weight gain in n-3/- mice. n-3/- FOS mice exhibited the higher body weight gain compared with the three other groups (Table 1).

**Table 1:** Energy intake and energy efficiency

	n-3/+	n-3/+ FOS	n-3/-	n-3/- FOS
Body weight gain (g)	-0.634 ± 0.512	0.443 ± 0.398	1.072 ± 0.157*	1.858 ± 0.197^#^
Energy intake (kcal)	968.7 ± 63.6	957.6 ± 8.1	885.6 ± 4.4	822.6 ± 14.3
Energy efficiency (g/100 kcal)	0.015 ± 0.114	0.184 ± 0.167	0.484 ± 0.071*	0.900 ± 0.093^#^

Energy efficiency is calculated as the ratio between body weight gain and caloric intake during the 24 days of treatment.

Data are the mean ± SEM.

*: mean values significantly different from n-3/+ mice (P < 0.05, Student's *t*-test).

^#^: mean values significantly different from n-3/- mice (P < 0.05, Student's *t*-test).

-3/+: mice fed with a control diet (n = 9), n-3/+ FOS: mice fed a control diet and supplemented with a prebiotic for 24 days (n = 10), n-3/-: mice fed an n-3 PUFA-depleted diet (n = 12), n-3/- FOS: mice fed an n-3 PUFA-depleted diet and supplemented with a prebiotic for 24 days (n = 12).

The results have been partly reported in reference 20.

Energy intake during the treatment was similar between groups. However, the energy efficiency, calculated as the ratio between body weight gain and energy intake during the treatment, was higher in n-3/- mice compared with n-3/+ mice. FOS supplementation increased energy efficiency in n-3/- mice. As observed for the body weight gain, n-3/- FOS mice exhibited the higher energy efficiency compared with the other groups (Table 1).

No significant modification in total adipose tissue weights was observed between groups However, we observed the highest visceral and subcutaneous fat mass in n-3/- FOS mice (Table 2).

**Table 2:** Adipose tissue weight

	n-3/+	n-3/+ FOS	n-3/-	n-3/- FOS
VAT (g/100 g body weight)	0.66 ± 0.11	0.76 ± 0.06	0.66 ± 0.02	0.80 ± 0.02^#^
OAT (g/100 g body weight)	0.81 ± 0.14	1.13 ± 0.07	1.02 ± 0.08	0.99 ± 0.08
SAT (g/100 g body weight)	1.21 ± 0.16	1.27 ± 0.08	1.41 ± 0.06	1.45 ± 0.12

Total AT (g/100 g body weight)	2.69 ± 0.38	3.06 ± 0.22	3.09 ± 0.13	3.17 ± 0.22

VAT; visceral adipose tissue, OAT; ovarian adipose tissue, SAT; subcutaneous adipose tissue, AT; adipose tissue

Data are the mean ± SEM.

*: mean values significantly different from n-3/+ mice (P < 0.05, Student's *t*-test).

^#^: mean values significantly different from n-3/- mice (P < 0.05, Student's *t*-test).

-3/+: mice fed with a control diet (n = 9), n-3/+ FOS: mice fed a control diet and supplemented with a prebiotic for 24 days (n = 10), n-3/-: mice fed an n-3 PUFA-depleted diet (n = 12), n-3/- FOS: mice fed an n-3 PUFA-depleted diet and supplemented with a prebiotic for 24 days (n = 12).

The results have been partly reported in reference 20.

#### Prebiotic intervention inverts fatty acid catabolism in the liver of n-3/- mice, without lessening hepatic TG accumulation

The liver weight was higher in n-3/+ mice compared with n-3/+ FOS and n-3/- mice (Table 3). n-3/- mice exhibited hepatic TG accumulation (Table 3), which mainly consisted of macrovesicular steatosis spread in all zones of the liver lobule, compared to n-3/+ mice (data not shown). FOS treatment had no significant effect on hepatic triglycerides content in either n-3/+ or n-3/- mice (Table 3), whereas it decreases lipid vesicles size in the liver tissue of n-3/- mice (data not shown). The hepatic cholesterol content was similar between the four groups (Table 3).

**Table 3:** Liver weight, triglycerides and cholesterol content and plasma triglycerides and cholesterol concentration

	n-3/+	n-3/+ FOS	n-3/-	n-3/- FOS
Liver weight (g/100 g body weight)	4.50 ± 0.15	3.80 ± 0.11*	3.97 ± 0.08*	3.89 ± 0.05
Hepatic triglycerides content (nmol/mg proteins)	97.6 ± 10.0	115.3 ± 11.9	159.3 ± 15.4*	176.6 ± 14.5
Hepatic cholesterol content (nmol/mg proteins)	37.1 ± 2.6	43.3 ± 1.9	42.6 ± 1.8	40.8 ± 2.2
Plasma triglycerides concentration (mM)	0.41 ± 0.4	0.34 ± 0.03	0.35 ± 0.04	0.35 ± 0.03
Plasma cholesterol concentration (mM)	1.04 ± 0.05	1.00 ± 0.02	1.49 ± 0.04*	1.37 ± 0.08

Data are the mean ± SEM.

*: mean values significantly different from n-3/+ mice (P < 0.05, Student's *t*-test).

^#^: mean values significantly different from n-3/- mice (P < 0.05, Student's *t*-test).

-3/+: mice fed with a control diet (n = 9), n-3/+ FOS: mice fed a control diet and supplemented with a prebiotic for 24 days (n = 10), n-3/-: mice fed an n-3 PUFA-depleted diet (n = 12), n-3/- FOS: mice fed an n-3 PUFA-depleted diet and supplemented with a prebiotic for 24 days (n = 12).

**Figure 4:**
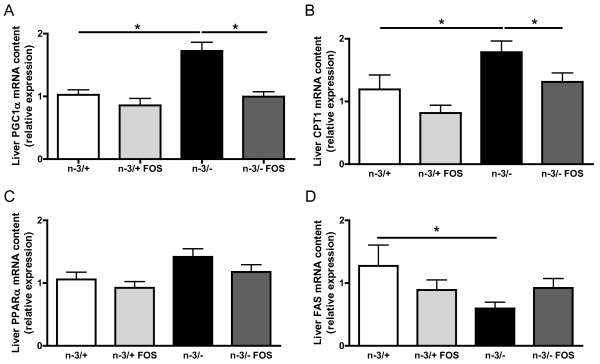
**Hepatic mRNA pattern**. (A) PGC1α: peroxisome proliferator-activated receptor gamma coactivator α, (B) CPT1: carnitine palmitoyl transferase 1, (C) PPARα: peroxisome proliferator-activated receptor α, (D) FAS: fatty acid synthase. Results are expressed as relative expression. Data are the mean ± SEM. *: mean values significantly different (P < 0.05, Student's *t*-test). -3/+: mice fed with a control diet (n = 9), n-3/+ FOS: mice fed a control diet and supplemented with a prebiotic for 24 days (n = 10), n-3/-: mice fed an n-3 PUFA-depleted diet (n = 12), n-3/- FOS: mice fed an n-3 PUFA-depleted diet and supplemented with a prebiotic for 24 days (n = 12). The results have been partly reported in reference 20.

The results have been partly reported in reference 20.

n-3/- mice exhibited a modified liver mRNA content of key factors involved in lipid metabolism as compared to n-3/+ mice. The expression of PGC1α, a transcription factor involved in β-oxidation, and CPT1, the rate limiting enzyme for β-oxidation, was increased in n-3/- mice compared with n-3/+ mice (Figure 4A and 4B). The hepatic mRNA content of PPARα, another transcription factor involved in β-oxidation, tended to be higher in n-3/- mice compared with n-3/+ mice (Figure 4C). FOS supplementation had no effect on the expression of PGC1α, CPT1 and PPARα in n-3/+ mice (Figure 4A, B and 4C), whereas it reduced hepatic mRNA level of PGC1α, CPT1 and PPARα in n-3/- mice to values similar to the ones found in n-3/+ mice (Figure 4A, B and 4C). n-3/- mice exhibited a lower expression of FAS, the rate-limiting enzyme for fatty acids synthesis, compared with n-3/+ mice, an effect which was not significantly modified by FOS treatment (Figure 4D).

There were no differences between groups in plasma TG concentration (Table 3). n-3/- mice exhibited higher plasma cholesterol concentration compared with n-3/+ mice, while FOS supplementation has no effect in either n-3/+ or n-3/- mice (Table 3).

#### Prebiotic supplementation reduces fasting glycemia but does not improve insulin response to an oral glucose tolerance test in n-3/- mice

**Figure 5:**
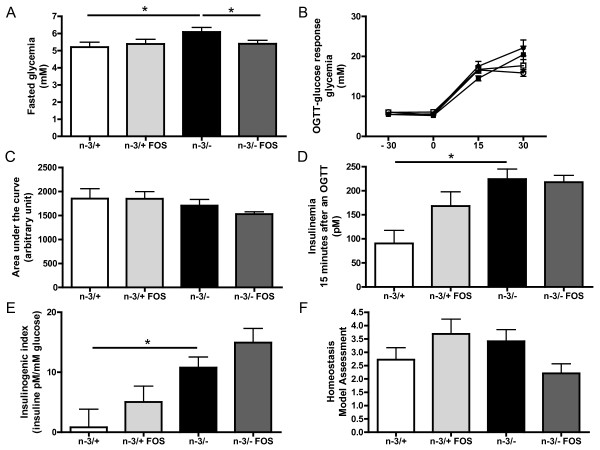
**Glucose homeostasis**. (A) fasted glycemia (mM), (B) glycemia response (mM) after a glucose challenge in n-3/+ mice (closed squares), in n-3/+ FOS mice (closed circles), in n-3/- mice (open squares) and in n-3/- FOS mice (open circles), (C) area under the curve (arbitrary unit): glycemia response after an oral glucose load, (D) insulinemia 15 minutes after an oral glucose load, (E) insulinogenic index 15 minutes after an oral glucose load, (F) homeostasis model assessment of insulin resistance. Data are the mean ± SEM. *: mean values significantly different (P < 0.05, Student's *t*-test). -3/+: mice fed with a control diet (n = 9), n-3/+ FOS: mice fed a control diet and supplemented with a prebiotic for 24 days (n = 10), n-3/-: mice fed an n-3 PUFA-depleted diet (n = 12), n-3/- FOS: mice fed an n-3 PUFA-depleted diet and supplemented with a prebiotic for 24 days (n = 12). The results have been partly reported in reference 20.

n-3/- mice exhibited higher fasting glycemia compared with n-3/+ mice (Figure 5A). There was no difference between groups in fasting insulinemia, measured 30 minutes before the glucose challenge (data not shown). After an oral glucose tolerance test, the glycemic excursion and the area under the curve for glycemia were similar in all groups (Figure 5B and 5C). However, the level of insulin measured 15 min after the glucose load was much higher in n-3/- mice compared with n-3/+ mice (Figure 5D) leading to an increased insulinogenic index. FOS supplementation decreased fasting glycemia in n-3/-mice but not in n-3/+ mice (Figure 5A). The insulinogenic index, calculated from insulin secretion 15 minutes after the glucose load, was higher in n-3/- than in n-3/+ mice, whereas FOS had no significant effect (Figure 5E). The homeostasis model assessment of insulin resistance suggests that FOS supplementation improves insulin sensitivity measured in the fasting state in n-3/- mice, even if it was not statistically significant (p = 0.06) (Figure 5F).

## Discussion

Mice fed with a diet depleted in n-3 PUFA for two generations exhibit several metabolic alterations compared to mice fed a diet with an adequate content in n-3 PUFA. Those alterations are: a lower body weight, a higher fasting glycemia and hepatic accumulation of triglycerides leading to macrovesicular steatosis. Liver mRNA expression of key factors involved in lipid metabolism could not explain this steatosis since markers involved in β-oxidation seemed to be promoted while markers reflecting the lipogenic pathway decreased [21].

Some recent studies have highlighted the important role of gut microbiota in the regulation of energy metabolism [22,23]. In our previous study, a decrease in caecal tissue and content weights was observed in n-3/- mice, despite the roughly similar total content in fibre between the two diets, which suggest a lower metabolic activity of the gut microbiota [21]. A possible relation between n-3 PUFA level in the diet and gut bacteria composition has been suggested by few studies. In fact, a study conducted in rats has described a decreased content of *bacteroides *after fish oil consumption, which is rich in long chain n-3 PUFA [41]. The inhibitory effect of n-3 PUFA on *bacteroides *was confirmed by other studies [42]. Surprisingly, in our study, n-3/- mice presented a slight decrease in bacteroides. On the other hand, a study carried out in fish has reported an increase content of lactobacilli under n-3 PUFA consumption and a lower content under n-6 PUFA consumption [43]. Coherently, we show here in mice that the content of lactobacilli in n-3/- mice is lower than in n-3/+ mice. Until now, the implication of such changes on the metabolic alterations remains unknown.

Of note, n-3/- mice exhibited no inflammatory state (data not shown), despite the fact that n-3 PUFA are well known modulators of the inflammatory process [44]. The higher bifidobacteria content observed in the caecum of n-3/- mice could be involved in the protection against the inflammation expected under n-3 PUFA depletion, as they have been shown to correlate inversely with inflammation and endotoxaemia in mice [35].

Lactobacilli are considered as important genera of saccharolytic bacteria, able to promote the release of short chain fatty acids upon fermentation and thereby to spare energy coming from the diet [33]. We can propose that the decrease in body weight, as well as the hepatic catabolic state observed in n-3/- mice, could be partly due to the lower global fermentation, estimated by the measurement of caecum weight. The fermentation of prebiotics FOS produces short chain fatty acids in the caeco-colon that provides energy supply for host, with an equivalent of 1 to 1.5 kcal per g of diet [45]. The gut microbiota can also modulate host energy metabolism by changing the expression of factors controlling fat storage, such as the fasting-induced adipocyte factor [22]. However, this effect does not seem to contribute to the metabolic effect of FOS in this case, since the level of expression of FIAF-which acts as a regulator of lipoprotein lipase activity- was unchanged in the visceral adipose tissue or liver of mice in the different groups (data not shown).

The restoration, by FOS feeding, of the caecal content and the increase in lactobacilli, together with the maintenance of high counts of bifidobacteria, as previously shown in other models [27,35], can contribute to increase both energy efficiency and body weight gain. It may also contribute to restore an adequate anabolic/catabolic balance in n-3/- mice, since prebiotic supplementation in n-3/- mice leads to a restoration of key hepatic factors involved in the fatty acid catabolism (CPT1, PGC1α and PPARα expression). Several studies have reported a lower steatosis linked to a decrease in lipogenesis in rodents under FOS supplementation [46,47]. On the contrary, FAS expression, the key enzyme involved in fatty acid synthesis, which is lower in the liver of n-3/- mice, was not modified by FOS treatment. It would suggest an improvement of global energy homeostasis in the liver of n-3 PUFA depleted mice supplemented with FOS.

The beneficial effects of FOS supplementation (decrease in hyperglycemia, improvement of insulin sensitivity) in animal models of obesity are reported to be partly linked to an increased expression of proglucagon and production of glucagon-like peptide-1 by endocrine L-cells present in the colon [48]. We have shown here that n-3/- mice exhibited a lower colonic content of proglucagon mRNA compared with n-3/+ mice, whereas FOS supplementation, in both n-3/- and n-3/+ mice, increased the expression of this gene. This could contribute to the improvement of insulin sensitivity observed in the fasting state in n-3/- mice.

One potential mechanism involved in the changes in bacteria composition linked to n-3 depletion, as well as some metabolic effects of this diet, could be the difference in the nutritional composition of the diets. It should be noted that the n-3/- diet, used by other authors [20,49], was richer in saccharose than the n-3/+ diet. It is however unlikely that this has an influence: feeding rats with a diet containing 15% of saccharose had only little effects on gut microbiota as compared to a non supplemented diet [50]. The increased content of saccharose in the diet could neither explained the effect on liver triglycerides accumulation [51] and on the higher fasting glycemia [52].

## Conclusions

In conclusion, a low n-3 PUFA dietary consumption is associated to metabolic alterations that target the liver, leading to steatosis. This long term nutritional depletion is associated with a decrease in global fermentation and with modifications of gut microbiota, characterized by a huge drop of lactobacilli and, unexpectedly, an increase in bifidobacteria. Interestingly, a relatively short treatment with prebiotics can promotes energy harvesting and reverses the decrease in caecal lactobacilli, the higher fasting glycemia and the higher expression of key factors involved in hepatic fatty acid catabolism. The relevance of gut microbiota modulation in the metabolic disorders associated with nutritional unbalance of fatty acids is a novel concept that merits attention in further studies.

## List of abbreviations

CPT1: carnitine palmitoyl transferase 1; FAS: fatty acid synthase; FOS: fructooligosaccharide; PGC1α: peroxisome proliferator-activated receptor gamma coactivator α; PPARα: peroxisome proliferator-activated receptor α; PUFA: polyunsaturated fatty acid, TG: triglyceride.

## Competing interests

The authors declare that they have no competing interests.

## Authors' contributions

BDP, AMN, PDC and NMD: conceived and designed the experiments. BDP, LP, FCDB, FMS, MH, and YAC: performed the experiments. BDP, AMN, PDC and NMD analyzed and interpreted the data. BDP, AMN, PDC and NMD wrote the manuscript. All Authors read and approved the final manuscript.
